# Camelina straw as an eco-friendly forage alternative for finishing lambs: impacts on performance, rumen function and behavior

**DOI:** 10.1016/j.vas.2025.100500

**Published:** 2025-08-21

**Authors:** Fatemeh Rezanejad, Seyed Mehdi Ghoreishi, Shahryar Kargar, Mohammad Javad Abarghuei, Ali Kahyani

**Affiliations:** a Department of Animal Science, School of Agriculture Shiraz University, Shiraz 71441–65186, Iran; b Department of Animal Science, College of Agriculture, Isfahan University of Technology, Isfahan 84156-83111, Iran; c Animal Science Research Department, Fars Agricultural and Natural Resources research and Education Center, Agricultural Research, Education and Extension Organization (AREEO), Shiraz, Iran

**Keywords:** Alfalfa replacement, Camelina straw, Digestibility, Feeding behavior, Lamb growth, Rumen ecology

## Abstract

The increasing frequency of droughts and water scarcity has intensified interest in drought-tolerant plants and their by-products as sustainable feed alternatives for ruminants. This study assessed the partial replacement of alfalfa hay (AH) with camelina straw (CS) at 120 (CS12) and 240 (CS24) g/kg of DM in diets of Grey Shirazi lambs (*n* = 24, average body weight = 22.7 ± 1.7 kg). No significant differences were observed in dry matter intake (DMI), final body weight (FBW: 40.4, 38.8, 40.0 kg), average daily gain (ADG: 285, 260, 278 g/d), or feed conversion ratio (FCR: 5.2, 5.3, 5.1) across treatments. Linear reductions occurred in OM and NDFom digestibility (*P* < 0.05), with no effects on other nutrients. Ruminal pH, NH_3__—_N, and volatile fatty acids (VFAs) profiles were not significantly altered, except for acetate and isovalerate concentrations, which increased linearly. Partial replacement of AH with CS increased ruminating and total chewing times (*P* < 0.01), due to higher physically effective fiber intake, without altering meal frequency or duration. These results suggest that camelina straw can be used as a partial AH substitute in lamb finishing diets up to 240 g/kg DM without compromising lamb performance, despite modest reductions in fiber digestibility and increased chewing activity. Future studies should explore behavioral and microbial ecological shifts in the rumen to better understand the mechanisms involved.

## Highlights

• Camelina straw (CS) can replace alfalfa hay in lamb diets up to 240 g/kg DM without compromising growth performance or feed efficiency.
• Dry matter intake, ADG, and FCR remained unaffected by CS inclusion.
• Organic matter and NDF digestibility decreased linearly with higher CS levels, likely due to increased lignin content.
• Ruminal pH and ammonia-N concentrations were stable across diets, indicating maintained rumen fermentation conditions.
• Camelina straw inclusion increased acetate production and extended ruminating time, suggesting enhanced physical effectiveness of fiber for rumen health.

## 1 Introduction

In recent years, the management and availability of feed resources, especially agricultural by-products, have become critical factors in enhancing animal production efficiency and achieving agricultural sustainability. Utilizing these by-products in livestock diets promotes a circular agri-food system, contributing to affordable and nutritious food production (Gatto et al., 2024; Ghoreishi et al., 2021; Mohammadi et al., 2024). Conventional forages such as alfalfa, corn silage, and cereal straw have traditionally formed the backbone of ruminant nutrition, with their selection influenced by nutritional quality, cost, and environmental constraints. However, global challenges including climate change, increasing forage costs, and water scarcity are severely impacting forage availability and sustainability, forcing a need to explore drought-resistant alternative feed resources.

Severe droughts, such as those recently experienced in Iran, have caused substantial reductions in crop yields and forage availability, thereby affecting the socioeconomic welfare of agricultural communities (Kheyruri et al., 2023). These hydrological drought events have highlighted the urgent need for forage alternatives that can thrive under water-limited conditions and support sustainable animal production.

*Camelina sativa,* a member of the *Brassicaceae* family, is a drought-tolerant crop with promising agronomic traits including low water requirements, adaptability to marginal and saline soils, and resistance to harsh climatic conditions (Alberghini et al., 2022; Paula et al., 2019). Camelina straw, a by-product of the crop, has similar nutritional properties to other energy crop straws and presents a viable and eco-friendly forage alternative, especially relevant for intensive fattening systems facing feed resource limitations (Owczuk & Kołodziejczyk, 2011). Initial investigations into the straw of various *Camelina sativa* varieties indicate that its nutritional composition includes DM (89.69–90.76 %), CP (5.49–9.44 %), NDF (76.19–83.65 %), and digestibility (31.11–35.55 % DM). Despite its potential benefits, research on camelina straw as a feed resource remains scarce, with previous studies focusing mainly on seeds and meals.

This study aims to evaluate the partial replacement of conventional forage—alfalfa hay—with camelina straw in the diets of Grey Shirazi finishing lambs. It investigates camelina straw’s effects on feed intake, growth performance, nutrient digestibility, ruminal fermentation, and ingestive behavior. By addressing the unique suitability of camelina straw in confined fattening systems under drought-prone conditions and connecting local findings with global feed sustainability challenges, this research contributes new scientific insights for environmentally sustainable and economically viable lamb production.

We hypothesized that camelina straw could maintain lamb performance without adverse effects on rumen fermentation, offering a cost-effective and sustainable alternative in regions impacted by forage scarcity and climate variability.

## 2 Materials and methods

### 2.1 Ethics statement

The experiment was conducted at the Animal Research Station of the College of Agriculture, Shiraz University, Shiraz, Iran (29°44′ N, 52°59′ E; altitude: 1641 m above sea level). All experimental procedures involving animals were conducted in accordance with the guidelines and regulations for the care and use of animals approved by the Iranian Council of Animal Care and adhered to the ARRIVE guidelines for reporting in vivo experiments (IACUC#2019/03.01). At the end of the study, a licensed veterinarian confirmed that the animals exhibited no signs of distress or adverse effects resulting from the experimental procedures.

### 2.2 Plant preparation

The cultivation of camelina was carried out in a farm located at the Zarghan Agriculture and Natural Resources Research Station in Shiraz (Southwest Iran, 29°76′ N, 52°71′ E, 1612 m above sea level). After harvesting the seeds operation, the collected CS was transported to the animal farm in Shiraz (30°07′ N, 53°14′ E, 1700 m above sea level) and chopped into 3 - 5 cm length.

### 2.3 Animal and diets

Twenty-four male Grey Shirazi fattening lambs (mean body weight, 22.7 ± 1.7 kg and mean age 105.7 ± 1.5 days) were randomly assigned to one of the following three ration groups (*n* = 8) which differed in the rate of CS replacement: control diet (without CS); CS12, diet containing 120 g/kg DM CS; and CS24, diet containing 240 g/kg DM CS (Table 1). The experimental diets were adjusted based on the lambs’ nutritional needs, based on NRC (2007). The diets were balanced for 14.5 % CP and 2.7 ME (Mcal/kg DM), with a forage-to-concentrate ratio of 30:70 % DM (Table 1). The study was conducted for 77 days, with a 14-day adaptation period. Before the start of the experiment, the lambs were sheared, antiparasitic drugs were administered and an enterotoxemia vaccine was injected. The lambs were fed twice daily at 08:00 and 16:00. Throughout the experimental period, the lambs were individually kept in separate pens (1.5 *m* × 1.5 m) and had access to sufficient drinking water. To ensure that 10 % of the total feed offered remained as leftovers, the remaining feed was gathered and weighed daily to estimate and adjust the DMI.

**Table 1:** Ingredient, chemical composition, particle size distribution and physical effective fiber content of the basal experimental diets.

Items	Diets^a^		
	Control	CS12	CS24
Ingredient (g*/*kg DM)^b^			
Alfalfa forage	300.0	180.0	60.0
Camelina straw, chopped	-	120.0	240.0
Barely grain	380.2	380.0	361.5
Soybean meal	66.0	111.2	155.7
Wheat bran	233.1	187.9	160.4
Salt	5.0	5.0	5.0
Mineral/vitamin premix	5.1	5.1	5.1
Sodium bicarbonate	10.4	10.4	10.4
Oystershell	0.2	0.4	1.9
Chemical composition (g*/*kg DM)			
DM	942.3	944.1	946.0
CP	145.0	145.0	145.0
Ash	73.5	63.5	53.8
EE	24.6	23.4	22.4
NDFom	337.4	347.5	361.7
Calcium	5.7	5.7	5.6
Phosphorus	4.8	4.6	4.5
Metabolizable energy (Mcal/kg DM)	2.7	2.7	2.7
Particle size distribution and physical effective fiber (% DM)^c^			
19 mm	2.13	4.24	10.97
8 mm	9.00	15.3	24.42
1.18 mm	32.78	33.66	31.73
Pan	56.09	46.87	32.88
Pef8	11.13	19.47	35.39
Pef_1.18_	43.91	53.13	67.12
peNDF_8_	3.76	6.76	12.80
peNDF_1.18_	14.82	18.46	24.27

a Control, control diet (without camelina straw); CS12, diet containing 120 g/kg DM camelina straw; CS24, diet containing 240 g/kg DM camelina straw.

b Mineral/vitamin premix (g/kg): vitamin A, 8000 IU, 2000 IU and vitamin E 50 mg, vitamin D3, Fe 80 mg, Cu 15 mg, Mn 50 mg, Co 0.3 mg, Zn 70 mg, I 0.6 mg, Se 0.3 mg. CP, crude protein; EE, ether extract; NDFom, ash-free NDF.

c Particle size distribution analysis using the Penn State Particle Separator ( % of DM retained on sieve) and the physically effective factor (pef) was calculated based on the proportion of DM retained on specific sieves. (Kononoff et al., 2003; Lammers et al., 1996).

### 2.4 DMI, growth performance, nutrient digestibility and rumen parameters

The offered diet and orts were weighed daily to calculate the daily feed intake. Samples were taken from each diet and orts and stored at −20 °C for further tests. The lambs were weighed every 20 days before morning feeding. The ADG and FCR were determined using the following equations:

$$\begin{array}{c} \text{ADG}=\left[\left(\text{Final}\text{body}\text{weight}-\text{Initial}\text{body}\text{weight}\right)/\text{Number}\text{of}\text{days}\right]\\ \text{FCR}=\left[\text{DMI}/\text{ADG}\right] \end{array}$$

The apparent digestibility of nutrients was determined by the total fecal collection method (Givens et al., 2000). On day 63 (after completing the fattening period to ensure stable intake patterns and rumen function), 5 lambs per treatment were transferred to individual metabolism crates with fecal and urine collectors and continued to be fed the experimental diets (Nargeszadeh et al., 2024). Following a week for adaptation to the metabolism crates, sample collection was carried out for 6 consecutive days (Obeidat, 2017). Each day before the morning feeding, total fecal excretion from each lamb were collected, weighed, and then thoroughly homogenized. The amounts of feed offered and feed refusal were also recorded daily. Representative sub-samples were collected from feces, feed, and refusal. At the end of the 6-day collection period, daily samples per lamb were pooled, thoroughly homogenized, and stored frozen at −21 °C for subsequent analyses. The apparent digestibility was determined using the following equation:

Apparentdigestibility(%)=[(Nutrientinfeed−Nutrientinfeces)/Nutrientinfeed]×100

Ruminal fluid was collected via stomach tube on days 61, 3 h after morning feeding. This timing was selected to ensure that the animals were fully adapted to their rations and that ruminal microbial populations had reached a steady state. pH of the rumen liquor was recorded immediately by a portable digital pH meter (Sartorius PT-10; Sartorius AG, Göttingen, Germany). Ruminal fluid was filtered through 4 layers of cheesecloth and then 2 ml of 25 % metaphosphoric acid was added to 8 ml of the filtered liquor. The rumen fluid samples were kept at −20 °C until analysis for NH_3__—_N and VFA. Ammonia-N was determined according to the colorimetric method of Broderick and Kang (1980). The VFAs were determined by gas chromatography (Chrompack, model CP-9002; Chrompack International BV, Middelburg, the Netherlands) with a fused silica column (50 *m* × 0.32 mm; CP-Wax Chrompack Capillary Column; Varian Inc., Palo Alto, CA). The internal standard was crotonic acid and the carrier gas was nitrogen. The initial and final temperatures of the oven were 55 °C and 195 °C, respectively, and the detector and injector temperatures were set at 250 °C.

### 2.5 Particle size determination and ingestive behavior

Frozen diet samples and refusals were thawed overnight at room temperature for particle size distribution analysis using the Penn State Particle Separator (PSPS; Kononoff et al., 2003). The DM content of each sieved fraction was determined by oven-drying. The physically effective factor (pef) was calculated based on the proportion of DM retained on specific sieves. For pef_8_, the proportion of DM retained on the 8-mm and 19-mm sieves was used (Lammers et al., 1996). Similarly, pef_1.18_ was calculated as the proportion of DM retained on the 1.18-mm, 8-mm, and 19-mm sieves (Kononoff et al., 2003). To determine the physically effective NDF (peNDF), the pef_1.18_ and pef_8_ values were multiplied by the dietary NDF content. The resulting values were designated as peNDF_>1.18_ (particles >1.18 mm) and peNDF_>8_ (particles >8 mm).

Behavioral observations followed the methodology described by Martin and Bateson (1993). On day 58 of the experimental period, feeding behavior was assessed by recording the time lambs spent eating, ruminating, and idling. Day 58 was chosen so that animals were fully adapted to the diets and established feeding behaviors. Continuous 24-hour observations were conducted at 5-minute intervals. To minimize disturbance, four trained observers rotated shifts while maintaining a non-intrusive position. Total chewing time was calculated as the sum of eating and rumination durations. Furthermore, each feeding activity was expressed relative to the intake of DM, NDF, forage NDF (fNDF), and peNDF, as described by Kahyani et al. (2022). Nighttime observations were conducted under artificial lighting, ensuring that animals had adapted to the conditions beforehand.

### 2.6 Laboratory measurements

Dry matter content of CS, diets, and orts samples was determined by oven drying at 100 °C for 24 h (AOAC, 2000; method 925.40). Ash content was determined by burning in an electric furnace at 550 °C overnight (AOAC, 2000; method 942.05). The OM content was calculated as the difference between 100 and the ash percentage. The EE was analyzed according to standard method (AOAC, 2000; method920.39). The NDFom was determined without sodium sulfite in neutral detergent (ND) according to Van Soest et al. (1991). Nitrogen was measured using the Kjeldahl method (AOAC, 2000; method 955.04). The non-fibrous carbohydrate (NFC) was calculated according to NRC (2007) as: NFC g/kg DM = 1000 – (CP g/kg DM + NDFom g/kg DM + EE g/kg DM + Ash g/kg DM).

### 2.7 Statistical analysis

The experiment followed a completely randomized design. Data were analyzed using the MIXED procedure of SAS (SAS Institute Inc., 2002). The statistical model for growth performance, digestibility, ruminal parameters, and ingestive behavior was:

$$Y_{ij}=\mu +Ti+\beta \left(IBW_{j}-\bar{IBW}\right)+\varepsilon ij$$

Where *Yij* is the dependent variable for the *j*-th lamb in the *i* th treatment, *μ* is the overall mean, *Ti* is the fixed effect of the *i* th dietary treatment (*i* = Control, CS12, CS24), *β* is the linear regression coefficient for the covariate initial body weight, IBW*j* is the initial body weight of the *j*-th lamb, $\bar{IBW}$ is the overall mean initial body weight, and *εij* is the residual error.

For repeated measures data (DMI - measured over time), the model included the repeated effect of time and an autoregressive [AR(1)] covariance structure was selected based on the lowest Akaike's Information Criterion (AIC). Polynomial contrasts were applied to evaluate linear and quadratic responses to incremental levels of camelina straw inclusion (0, 120, and 240 g/kg DM). Least squares means were separated using the PDIFF option. Significance was declared at *P* < 0.05, and tendencies were noted at 0.05 ≤ *P* ≤ 0.10. All values are reported as least squares means ± standard error of the mean (SEM).

## 3 Results

### 3.1 Chemical composition of CS

The amounts of DM, CP, EE, ash, NDFom, ADFom, NFC, and ADL of camelina straw were 931.0, 22.0, 11.0, 40.0, 661.0, 513.0, 26.6, and 95.6 (g/kg DM) respectively.

### 3.2 DMI and growth performance

The results related to growth performance and feed intake are presented in Table 2. Replacing AH with CS has no effect on DMI, IBW, FBW, ADG and FCR (*P* > 0.05).

**Table 2:** DMI and performance of lambs fed diets containing different levels of Camelina straw.

Item	Diets			SEM	*P*-value		
	Control	CS12	CS24		Treatment	Linear	Quadratic
DMI (g/d)	1491.7	1372.2	1399.9	48.76	0.16	0.19	0.15
IBW (kg)	22.8	22.9	22.5	0.64	0.90	0.72	0.80
FBW (kg)	40.4	38.8	40.0	0.61	0.22	0.62	0.11
ADG (g)	285.4	260.3	278.3	10.21	0.22	0.59	0.10
FCR	5.2	5.3	5.1	0.18	0.67	0.55	0.51

Control, control diet (without camelina straw); CS12, diet containing 120 g/kg DM camelina straw; CS24, diet containing 240 g/kg DM camelina straw; DMI, dry matter intake; IBW, initial body weight; FBW, final body weight; ADG, average daily gain; FCR, feed conversion ratio (DMI/ADG). SEM, standard error of means.

### 3.3 Nutrient digestibility

The nutrient apparent digestibility in lambs fed camelina straw diets is illustrated in Table 3. The apparent digestibility of DM, CP, EE, and NFC were unaffected (*P* > 0.05) while digestibility of OM and NDFom decreased linearly by the level of replacement of CS.

**Table 3:** Apparent digestibility of nutrient in lambs diets containing different levels of camelina straw.

Item (%)	Diets			SEM	*P*-value		
	Control	CS12	CS24		Treatment	Linear	Quadratic
DM	73.8	72.0	70.2	1.26	0.20	0.08	0.99
OM	75.3	73.5	70.9	1.22	0.09	0.03	0.84
CP	80.6	79.7	83.8	1.34	0.13	0.13	0.17
EE	75.6	77.3	79.8	1.63	0.24	0.10	0.86
NDFom	57.6	57.1	49.8	2.28	0.07	0.04	0.25
NFC	88.0	86.6	88.5	1.03	0.42	0.71	0.22

Control, control diet (without camelina straw); CS12, diet containing 120 g/kg DM camelina straw; CS24, diet containing 240 g/kg DM camelina straw; SEM, standard error of means; CP, crude protein; EE, ether extract; NDFom, neutral detergent fiber, ash-free; ADFom, acid detergent fiber, ash-free; NFC, non-fiber carbohydrates.

### 3.4 Rumen parameters

The ruminal pH, NH_3__—_-N, total VFA and concentrations of propionate, isobutyrate, butyrate, valerate and the acetate/propionate ratio were not influenced by the level of replacement of CS (*P* > 0.05; Table 4). The concentration of the acetate and isovalerate in the rumen increased linearly in response to increasing dietary level of CS (*P* < 0.05).

**Table 4:** Ruminal fermentation parameters in lambs fed diets containing different levels of camelina straw.

Item	Diets			SEM	*P*-value		
	Control	CS12	CS24		Treatment	Linear	Quadratic
pH	6.91	6.71	6.82	0.16	0.67	0.56	0.48
NH_3__—_N (mg/dL)	9.14	8.47	8.87	1.21	0.89	0.86	0.65
Total VFA (mmol/L)	52.51	60.06	52.12	7.24	0.70	0.97	0.42
VFAs (mmol/100 mmol)							
Acetate	66.76	66.23	70.27	0.91	0.01	0.02	0.06
Propionate	21.46	19.58	17.56	1.69	0.27	0.12	0.97
Isobutyrate	0.44	0.56	0.75	0.10	0.18	0.08	0.80
Butyrate	10.49	12.60	10.37	1.28	0.34	0.94	0.16
Isovalerate	0.21	0.36	0.62	0.12	0.08	0.03	0.64
Valerate	0.64	0.67	0.43	0.11	0.29	0.25	0.32
Acetate/propionate	3.15^b^	3.40^ab^	4.20^a^	0.39	0.23	0.12	0.59

Control, control diet (without camelina straw); CS12, diet containing 120 g/kg DM camelina straw; CS24, diet containing 240 g/kg DM camelina straw; SEM, standard error of means.

### 3.5 Ingestive behavior

The physical characteristics of the experimental diets are presented in Table 1. Increasing the proportion of CS in the diets elevated the quantity of particles in the 8–19 mm and >19 mm length categories while reducing particles <1.18 mm (*P* < 0.05). Consequently, pef_8_ and pef_1.18_, and physically effective NDF (peNDF_>8_ and peNDF_>1.18_) increased with higher CS inclusion.

Nutrient and particle-size intake data are summarized in Table 5. Replacement of AH with CS did not DMI or total NDF intake (*P* > 0.05). However, forage NDF intake, peNDF_>8_, and peNDF_>1.18_ increased linearly with CS inclusion (*P* < 0.01). A linear rise was observed in the intake of larger particles (>19 mm and 8–19 mm; *P* < 0.01), whereas the intake of fine particles (<1.18 mm) declined linearly (*P* < 0.01).

**Table 5:** Nutrient intake of lambs fed diets containing different levels of camelina straw.

Item	Diets			SEM	*P*-value		
	Control	CS12	CS24		Treatment	Linear	Quadratic
Intake, kg/d							
DM	1750.2	1587.1	1681.7	80.83	0.36	0.53	0.20
NDF	590.6	551.5	608.2	27.79	0.34	0.64	0.17
Forage NDF	246.8	260.2	314.2	12.54	0.0015	<0.01	0.19
peNDF_>8_	65.7	107.4	215.2	5.245	<0.01	<0.01	<0.01
peNDF_>1.18_	259.3	293.0	408.2	14.10	<0.01	<0.01	0.03
Particle-size intake, kg/d							
19 mm	37.2	67.2	184.4	3.805	<0.01	<0.01	<0.01
19–8 mm	157.6	241.7	410.7	11.22	<0.01	<0.01	<0.01
1.18–8 mm	573.7	534.2	533.6	26.66	0.45	0.27	0.55
<1.18 mm	981.7	743.9	552.9	41.01	<0.01	<0.01	0.64

Control, control diet (without camelina straw); CS12, diet containing 120 g/kg DM camelina straw; CS24, diet containing 240 g/kg DM camelina straw. SEM, standard error of means.

Meal patterns (Table 6) were unaffected by dietary treatments. The meal number (14 meals/d), meal duration (19.9 min/meal), meal rate (6.43 g DM/min), and meal size (119 g DM) remained consistent across diets (*P* > 0.05). However, the rumination rate decreased linearly (*P* < 0.05), and Min/bout increased linearly (*P* < 0.01) with higher CS proportions.

**Table 6:** Meal patterns of lambs fed diets containing different levels of camelina straw.

Item	Diets			SEM	*P*-value		
	Control	CS12	CS24		Treatment	Linear	Quadratic
Eating							
Meals/d	14.5	14.8	13.6	0.91	0.59	0.48	0.47
Length, min/meal	17.3	21.1	21.5	2.08	0.26	0.14	0.52
Rate, g DM/min	7.6	5.6	6.0	0.74	0.15	0.13	0.20
Rumination							
Bouts/d	16.1	18.0	18.0	0.98	0.28	0.17	0.44
Min/bout	19.4	22.4	26.9	1.43	<0.01	<0.01	0.66
Rate, g DM/min	6.8	4.1	3.6	1.03	0.09	0.04	0.39

Control, control diet (without camelina straw); CS12, diet containing 120 g/kg DM camelina straw; CS24, diet containing 240 g/kg DM camelina straw. SEM, standard error of means.

Chewing activities (Table 7) revealed a linear increase in rumination time (*P* < 0.01) and total chewing time (*P* < 0.01) as CS inclusion increased. Eating time did not differ among treatments (mean: 279 ± 24.7 min/d; *P* = 0.21). When expressed per unit of intake, ruminating and chewing time per gram of DM or NDF rose linearly (*P* < 0.01), whereas chewing time per gram of peNDF_>8_ decreased linearly (*P* < 0.01).

**Table 7:** Time spent on eating, rumination, and chewing in lambs fed diets containing different levels of camelina straw.

Item	Diets			SEM	*P*-value		
	Control	CS12	CS24		Treatment	Linear	Quadratic
Eating time							
Min/d	247.50	309.29	283.13	24.73	0.21	0.29	0.16
Min/bout	17.26	21.06	21.55	2.08	0.26	0.14	0.52
Min/g DMI	0.14	0.20	0.17	0.02	0.15	0.39	0.08
Min/g NDF intake	0.43	0.59	0.47	0.06	0.17	0.63	0.08
Min/g forage NDF intake	1.02	1.25	0.91	0.14	0.21	0.52	0.10
Min/g peNDF_>8_	3.85	3.02	1.32	0.39	<0.01	<0.01	0.37
Ruminating time							
Min/d	313.75	402.86	484.37	30.65	0.01	<0.01	0.92
Min/bout	19.45	22.41	26.93	1.44	<0.01	<0.01	0.66
Min/g DMI	0.18	0.26	0.29	0.02	<0.01	<0.01	0.38
Min/g NDF intake	0.54	0.75	0.80	0.06	0.01	<0.01	0.32
Min/g forage NDF intake	1.29	1.58	1.55	0.14	0.25	0.17	0.33
Min/g peNDF_>8_	4.85	3.84	2.26	0.40	<0.01	<0.01	0.56
Total chewing time							
Min/d	561.25	712.14	767.50	37.17	<0.01	<0.01	0.30
Min/g DMI	0.33	0.46	0.46	0.03	0.01	0.01	0.11
Min/g NDF intake	0.97	1.34	1.27	0.10	0.03	0.03	0.09
Min/g forage NDF intake	2.32	2.83	2.46	0.22	0.24	0.64	0.11
Min/g peNDF_>8_	8.70	6.86	3.59	0.62	<0.01	<0.01	0.35

Control, control diet (without camelina straw); CS12, diet containing 120 g/kg DM camelina straw; CS24, diet containing 240 g/kg DM camelina straw. SEM, standard error of means.

## 4 Discussion

### 4.1 Chemical composition of CS

The amounts of DM, CP, EE, ash, NDFom, ADFom and ADL of camelina straw were 931.0, 22.0, 11.0, 40.0, 661.0, 513.0 and 95.6 (g/kg DM) respectively. Compared to wheat straw, as a common straw in ruminant feeding, camelina straw has higher amounts of ADFom, and ADL but lower amounts of ash, CP, EE and NDFom. There is very little research on the chemical composition of camelina straw. *In vitro* research, the straw characterization of nine new varieties of *Camelina sativa* (L.) Crantz was investigated. The amounts of DM, CP, EE, ash, NDFom and ADFom of camelina straws were determined as 896.9–907.6, 54.9–94.4, 4.7–11.2, 38.3–60.6, 761.9–836.5 and 581.1–638.5 g/kg DM, respectively (Montero-Muñoz et al., 2023).

### 4.2 DMI and growth performance

In the present study, DMI, FBW, FCR, and ADG was not affected by the addition of camelina straw. Various factors affect the consumption of feed by ruminants, including the animal and its physiological conditions, the composition of the diet, the palatability of the diet, the digestibility of the feed, the expansion of the rumen wall due to the filling properties of fibrous materials, the ratio of forage to concentrate, environmental conditions (temperature and humidity), and the presence of secondary metabolites and anti-nutritional compounds (Christodoulou et al., 2023; Kari et al., 2023; Van Soest, 1994).

The absence of an effect from partially replacing AH with camelina straw on DMI in the current study could be attributed to the similarity of the experimental diets, particularly with respect to their NDF content. As demonstrated by Obeidat et al. (2019), replacing half of the AH with wheat straw, which resulted in a 6 % increase in dietary NDF, did not significantly impact the DMI of lambs. Similarly, in the present study, substituting AH with 24 % camelina straw resulted in a 6.7 % increase in dietary NDF, aligning with the findings of the aforementioned study. However, a complete replacement of AH with wheat straw, which was associated with a 13 % increase in dietary NDF, significantly reduced DMI in lamb diets compared to diets containing only AH (Obeidat et al., 2019).

To date, no studies have examined the use of camelina straw in fattening lamb diets. However, research on similar alternative roughage sources may provide relevant comparisons. It has been reported that using a mixture of straw and AH (Obeidat et al., 2019) or straw combined with other forage sources (Awabdeh et al., 2022; Ismail & Obeidat, 2023) maintained the level of DMI in the diets of lambs. Obeidat et al. (2019) showed that replacing half of the AH with wheat straw had no significant effect on DMI, FBW, FCR, or ADG of lambs. Liu et al. (2023) investigated the effects of replacing AH with oat hay (33 %, 50 %, and 66 % DM) in fermented total mixed rations on lambs' DMI and growth performance. Both DMI and ADG were significantly higher in the 50 % treatment compared to 33 % and 66 %, although final body weight did not differ among treatments (Liu et al., 2023).

It has been reported that feed intake has a direct relationship with performance and weight gain (Ghoreishi et al., 2021; Khaldari & Ghiasi, 2018; Khurshid et al., 2023). Studies have shown that weight gain and daily gain can be influenced by increase dietary energy levels (Zhang et al., 2023). In the present study, the lack of a significant effect of different dietary camelina straw levels on ADG may be attributed to similar dry matter intake (DMI) across isoenergetic diets.

Similar patterns have been documented in comparable studies, where non-significant differences in ADG were consistently observed when DMI and digestibility parameters remained stable across treatments (Ismail & Obeidat, 2023; Nargeszadeh et al., 2024; Obeidat et al., 2019). Furthermore, consistent with Nasri et al. (2011), changes in lamb body weight under non-stressful conditions correlated strongly with feed intake and dry matter digestibility. In our study, neither parameter differed significantly between straw-supplemented and control diets (Table 2, Table 3), explaining the similar ADG observed across groups.

### 4.3 Nutrient digestibility

The digestibility of DM, CP, EE, and NFC remained unaffected by camelina straw inclusion among the experimental treatments (*P* > 0.05). Feedstuff digestibility is governed by multiple factors, including animal-related variables (species, age, health status, and rumen function), feed characteristics (chemical composition, physical structure, forage maturity), and management practices (harvesting stage, processing methods, feeding frequency, and levels). Individual animal variability and rumen conditions - particularly microbial populations and pH stability - further modulate digestibility (Li et al., 2022; McDonald et al., 2022). Reduced ruminal pH, for instance, can impair nutrient digestibility by altering rumen fermentation patterns (Li et al., 2022). Therefore, in the present experiment, the lack of difference in DM digestibility appears to be related to the uniformity of ruminal pH (Table 4) as well as the consistency in diet composition (Imani Rad et al., 2016; Papi et al., 2019).

The organic matter and NDF digestibility exhibited a linear decline with increasing camelina straw inclusion. The reduction in NDF digestibility observed in the CS24 diet may be attributed to the increased lignin content of camelina straw. Lignin is a key anti-nutritional factor that limits cell wall digestibility by physically impeding microbial access to structural polysaccharides. This steric hindrance prevents proper alignment of polysaccharidases with their substrates, inhibiting hydrolysis (Jung & Allen, 1995). Furthermore, lignin’s negative correlation with digestibility is not solely due to its concentration but also its cross-linking bonds with cell wall polysaccharides, which further restrict microbial degradation (Getachew et al., 2018). As plants mature, lignin deposition increases, leading to stronger lignocellulosic complexes and reduced digestibility (Van Soest, 1994).

In the present study, the higher lignin content and lignin composition in camelina straw—a byproduct of mature plants—likely contributed to the reduction in NDF digestibility. This aligns with previous studies where increased lignin levels were linked to diminished fiber digestibility, such as when alfalfa was replaced with sericea lespedeza in growing lambs (Mahachi et al., 2023) or when corn silage was substituted with sorghum silage in dairy cows (Lv et al., 2023). Similarly, Souza et al. (2024) observed reduced NDF digestibility in lambs fed diets supplemented with mushroom residues, which was attributed to the higher content of indigestible components such as ADF and lignin, as well as the lignocellulosic complex and lower fiber quality in the mushroom residues.

The observed decline in OM digestibility can be due to the decreased NDF digestibility and the higher lignin content in camelina straw. Organic matter digestibility has been shown to be affected by both the concentration of lignin and the extent of its cross-linking with structural polysaccharides. Increased lignin content consistently correlates with decreased plant OM digestibility (Getachew et al., 2018). In line with our results, the addition of a citrus pulp silage mixture to the diets of fattening lambs improved the digestibility of OM and NDF without affecting DM digestibility. This effect was attributed to the negative correlation observed between digestibility and ADF (Nargeszadeh et al., 2024). A similar pattern of decreased OM and NDF digestibility, without affecting DM digestibility, was reported when AH was replaced with Jerusalem artichoke aerial parts (Papi et al., 2019).

### 4.4 Rumen parameters

The inclusion of camelina straw at increasing levels did not significantly alter the ruminal pH or ammonia nitrogen (NH₃-N) concentrations, indicating that partial replacement of AH with camelina straw did not impair ruminal ammonia dynamics. Ruminal pH values varied from 6.7 to 6.9 (Table 4), which were within the optimum range (6.7 ± 0.5) for maintaining normal ruminal organisms (Van Soest, 1994). There is no study about CS feeding on ruminal pH and NH_3__—_N. In ruminants, rumen pH is prominently influenced by the dietary forage-to-concentrate ratio, diet composition and buffers such as bicarbonate, calcium carbonate, and magnesium oxide (Shen et al., 2023). Therefore, in the current research, there was no difference in nutritional conditions that would affect rumen pH.

In all experimental lambs, ruminal NH_3__—_N (8.47 - 9.14 mg/dL rumen liquor) was well above the minimum level required for optimal microbial growth (5.0 mg/dL) suggesting adequate microbial protein synthesis despite dietary changes (Sinclair et al., 1993). Various factors affect NH_3__—_N production in the rumen such as diet (rumen-degradable CP concentration of the diet, accessible energy available and plant secondary metabolites), species, and methodological differences (Eschenlauer et al., 2002; Kapp-Bitter et al., 2020). According to these factors, in the present research, we should not expect the amount of NH_3__—_N to be different between experimental lambs. The lack of differences in ruminal NH₃-N across groups also could be attributed to either similar CP intake (data not shown) or a dynamic equilibrium among microbial NH₃-N production, epithelial absorption, and ruminal outflow rate (Mahachi et al., 2023). The stability in NH_3__—_N concentrations across treatments is consistent with the apparent digestibility of CP, which also showed no significant differences among diets (Table 3) indicating that CS inclusion did not negatively impact protein utilization.

Volatile fatty acid production is influenced by dietary composition, including fiber quantity and type, ruminal pH, and microbial community dynamics (Calabrò et al., 2002; Li et al., 2019; Ma et al., 2024). Replacing AH with CS at dietary levels of 120 and 240 g/kg DM had no significant effect on total ruminal VFA concentration (Table 4). However, the proportions of acetate and isovalerate increased linearly with higher CS inclusion. This elevation in acetate aligns with the higher NDFom content of CS, as fibrous substrates promote acetate production through enhanced fibrolytic activity (Lourencon et al., 2024; Ma et al., 2024). The concurrent rise in isovalerate — a branched-chain fatty acid — is particularly noteworthy, as branched-chain fatty acids like isovalerate and isobutyrate serve as essential growth factors for cellulolytic bacteria. Specifically, genera such *as Ruminococcus* and *Fibrobacter* (key fibrolytic microbes) require branched-chain fatty acids to proliferate and degrade fiber. Thus, the increased isovalerate concentration likely stimulated cellulolytic populations, partially counteracting the inhibitory effects of lignin on fiber digestibility (Zhang et al., 2025). Future studies should directly investigate CS-induced shifts in rumen microbial composition to elucidate mechanistic links between dietary fiber alterations, microbial ecology, and VFA profiles.

The stability in total VFA concentrations despite higher acetate in the CS24 group may reflect compensatory mechanisms, such as numerically reduced propionate yield (*P* = 0.12) or lower NDF digestibility (49.8 % in CS24 vs. 57.6 % in control; *P* = 0.04). Similar patterns were reported in other studies, where high-fiber diets increased acetate without altering total VFA production (Gao et al., 2023; Li et al., 2019; Zhang et al., 2025). The lack of significant changes in propionate may be attributed to the diets’ similar NFC content (Owens et al., 2009), as fermentable carbohydrates (e.g., starch or sugars) likely maintained propionate synthesis pathways. Despite CS’s higher fiber content, adequate NFC availability may have prevented a pronounced decline in propionate concentration.

### 4.5 Ingestive behavior

The present study investigated the effects of replacing AH with Camelina straw (CS) on the chewing behavior of fattening lambs. Diets containing CS had a higher proportion of long particles (retained on the 19-mm and 8–19 mm sieves), pef_8_, pef_1.18_, peNDF_>8_, and peNDF_>1.18_ compared to diets without CS. These changes indicate alterations in the physical characteristics of the diets as CS inclusion increases.

Our results show that substituting CS for AH did not negatively affect DMI, meal number, meal length, meal rate, meal size, or eating time. However, ruminating time and total chewing time increased linearly. Particles retained on the 19-mm and 8–19 mm sieves are more effective in stimulating chewing than those retained on the 1.18-mm sieve (Heinrichs & Kononoff, 2002). This likely explains the elevated chewing activity observed in lambs fed CS diets, as these diets contained more particles retained on the 19-mm and 8–19 mm sieves (Table 1).

Analysis of DMI by particle size revealed that the consumption of particles measuring 19 mm, 8–19 mm, and 1.18–8 mm increased with higher levels of CS in the diet, which subsequently increased peNDF intake. Differences in diet particle size and peNDF intake among experimental lambs influenced rumination activity. This finding aligns with previous studies indicating that larger diet particle size and peNDF intake are key factors controlling rumination activity in ruminants (Mertens, 1997; Nasrollahi et al., 2016).

As expected, higher peNDF_>8_ concentrations increased total chewing time and ruminating time, reflecting the additional time required to masticate longer dietary particles. This is consistent with Kahyani et al. (2013), who reported simultaneous increases in peNDF_>8_ intake and total chewing time with higher peNDF_>8_ concentrations. Similarly, Nasrollahi et al. (2012) observed an increase in eating time associated with elevated peNDF concentrations. However, no significant differences in eating time were found across diets with varying peNDF concentrations, consistent with the similar DMI observed across treatments. These results suggest that ruminating time is more responsive to variations in peNDF_>8_ concentration than eating time in high-concentrate diets.

The linear increase in total chewing and ruminating time with higher CS levels is consistent with the greater proportions of particles retained on the 19-mm and 8–19 mm sieves, as well as higher value of pef_8_, pef_1.18_, peNDF_>8_, and peNDF_>1.18_ in CS diets. Intake of peNDF_>8_ emerged as the primary contributor to chewing time (Beauchemin & Yang, 2005). Lambs fed diets containing CS24 exhibited greater ruminating and chewing times due to higher forage NDF content, peNDF_>8_, peNDF_>1.18_, and large particle (>19 mm and 8–19 mm) intake.

Prolonged ruminating (by 54.4 % in CS24 vs. control; Table 7) stimulates saliva secretion, providing natural bicarbonate buffers that stabilize rumen pH and prevent acidosis—a critical advantage in high-concentrate feeding systems (Beauchemin & Yang, 2005). This mechanism explains the stable ruminal pH (6.7–6.9) despite shifts in VFA profiles. The extended chewing time induced by camelina straw’s physically effective fiber (>8 mm particles) is particularly beneficial in intensive systems, where acidosis risk necessitates robust dietary buffering. Conversely, in extensive systems, CS offers drought resilience, thriving on marginal lands with minimal irrigation—conditions unsuitable for alfalfa cultivation.

In summary, both peNDF_>8_ and peNDF_>1.18_ contribute to increased rumination time by providing physical stimuli that promote chewing and rumination (Heinrichs & Kononoff, 2002).

## 5 Conclusions

These findings demonstrate that camelina straw can replace alfalfa hay at inclusion levels up to 240 g/kg DM in fattening lamb diets without adverse effects on growth performance or feed efficiency. Replacing alfalfa hay with camelina straw at 240 g/kg DM reduced fiber digestibility by approximately 7.8 % but maintained ADG and rumen fermentation, supporting its use in drought-stressed regions. The inclusion of CS extended daily ruminating time by 54.4 %, enhancing rumen buffering through increased saliva production and linearly increased acetate production, indicating stimulated fibrolytic activity. This substitution is especially advantageous in intensive systems where concentrated feeding is practiced, as CS maintains rumen health via physically effective fiber. It further improves feed economic viability by valorizing low-input agricultural byproducts. Further research is recommended to explore its long-term effects on ruminal microbiota and overall health, as well as to investigate the implications of higher CS inclusion levels.

## Ethical statement

All experimental procedures involving animals were conducted in accordance with the guidelines and regulations for the care and use of animals approved by the Iranian Council of Animal Care and adhered to the ARRIVE guidelines for reporting in vivo experiments (IACUC#2019/03.01). At the end of the study, a licensed veterinarian confirmed that the animals exhibited no signs of distress or adverse effects resulting from the experimental procedures.

The authors confirm that the welfare of the animals was a primary consideration throughout the study, and all efforts were made to minimize discomfort and stress. No animals were subjected to unnecessary harm or procedures beyond standard husbandry practices.

## CRediT authorship contribution statement

**Fatemeh Rezanejad:** Methodology, Investigation, Conceptualization. **Seyed Mehdi Ghoreishi:** Writing – review & editing, Supervision, Formal analysis, Data curation, Conceptualization. **Shahryar Kargar:** Validation, Formal analysis. **Mohammad Javad Abarghuei:** Writing – original draft, Investigation. **Ali Kahyani:** Writing – original draft, Investigation.

## Declaration of competing interest

The authors wish to confirm that there are no known conflicts of interest associated with this publication and there has been no financial support for this work that could have influenced its outcome.
